# Interleukin 15 and Eotaxin correlate with the outcome of breast cancer patients vice versa independent of CTC status

**DOI:** 10.1007/s00404-020-05793-y

**Published:** 2020-09-14

**Authors:** Theresa Vilsmaier, Helene Hildegard Heidegger, Lennard Schröder, Elisabeth Trapp, Alaleh Zati zehni, Brigitte Rack, Wolfgang Janni, Sven Mahner, Tobias Weissenbacher, Udo Jeschke, Jan-Niclas Mumm, SUCCESS Study Group, Theresa Vilsmaier, Helene Hildegard Heidegger, Lennard Schröder

**Affiliations:** 1 Department of Gynecology and Obstetrics, Ludwig Maximilian University, Maistraße 11, 80337 Munich, Germany; 2 Department of Gynecology and Obstetrics, University Hospital Graz, Auenbruggerplatz 14/1, 8036 Graz, Austria; 3 Department of Gynecology and Obstetrics, University Hospital Ulm, Prittwitzerstr. 43, 89075 Ulm, Germany; 4 Department of Gynecology, Waldstraße 7, 82166 Gräfelfing, Germany; 5 Department of Urology, Ludwig Maximilian University, Marchioninistraße 15, 81377 Munich, Germany

**Keywords:** SUCCESS study, Breast cancer, Circulating tumor cells, Interleukin 15, Eotaxin

## Abstract

**Background:**

Circulating tumor cells (CTC) in the peripheral blood in women with breast cancer has been found to be an indicator of prognosis before the start of systemic treatment. The aim of this study is the assessment of specific cytokine profiles as markers for CTC involvement that could act as independent prognostic markers in terms of survival outcome for breast cancer patients.

**Methods:**

Patients selected for this study were defined as women with breast cancer of the SUCCESS study. A total of 200 patients’ sera were included in this study, 100 patients being positive for circulating tumor cells (CTC) and 100 patients being CTC negative. The matching criteria were histo-pathological grading, lymph node metastasis, hormone receptor status, TNM classification, and patient survival. Commercial ELISA with a multi cytokine/chemokine array was used to screen the sera for Interleukin 15 (IL-15) and eotaxin.

**Results:**

Statistically significant concentrations were exposed for IL-15 levels regardless of the CTC-Status, lymph node involvement, or hormone receptor status. Significantly enhanced serum IL-15 concentrations were observed in those patients with worse overall survival (OS) and disease-free survival (DFS). Elevated serum concentrations of IL-15 significantly correlate with patients diagnosed with Grade 3 tumor and worse OS. In contrast, patients with a Grade 3 tumor with a favourable OS and DFS demonstrated significantly decreased IL-15 values. The CTC negative patient subgroup with a favourable OS and DFS, showed statistically significant elevated eotaxin values.

**Conclusion:**

These findings suggest a potential functional interaction of increased IL-15 concentrations in the peripheral blood of patients with a worse OS and DFS, regardless of prognostic factors at primary diagnosis. The increased levels of the chemokine eotaxin in CTC negative patients and a favourable OS and DFS, on the other hand, suggest that the overexpression inhibits CTCs entering the peripheral blood, thus emphasizing a significant inhibition of circulation specific metastasis. To sum up, IL-15 could be used as an independent prognostic marker in terms of survival outcome for breast cancer patients and used as an early indicator to highlight high-risk patients and consequently the adjustment of cancer therapy strategies.

## Introduction

Breast cancer is the most common malignant tumor in women; yet invasive breast cancer is still considered one of the greatest challenges for experts to control and thereby improve the survival of patients [1]. The options for treatment of breast cancer comprise targeted therapies, chemotherapy, surgery, radiotherapy, aromatase inhibitors, and hormone-receptor modulators [2–4]. Even though the options have advanced extremely over the past years, the consistently high mortality rate, due to tumor metastasis to the lymph node and vital organs, remains [5]. Its mortality is mostly due to distant metastatic growth [5, 6].

The expression ‘liquid biopsy’ describes the observation and evaluation of treatment effectiveness in patients with breast cancer and refers to molecular analysis of the tumor’s genetic structures based on circulating genetic material in the peripheral blood derived from circulating tumor cells (CTCs) [7, 8]. The technology for detecting CTCs has progressed from simple cell counting into advanced molecular subtyping [9], and as a minimally invasive method it could be of tremendous importance in the future for early cancer identification and disease monitoring. The correlation between poor prognosis and the detection of CTCs before the start of systemic treatment has been described previously [10]. CTCs in the peripheral blood have been determined to be a prognostic marker for reduced disease-free survival (DFS), distant DFS and overall survival (OS) before the start of systemic treatment in both metastatic and non-metastatic breast cancer [10–14]. As CTCs are believed to originate from the primary tumor and to acquire genetic heterogeneity during evolution, CTC values also provide complementary information concerning the treatment response [14]. The SUCCESS study was one of the first trials to determine the prognostic association of CTCs with poorer survival in early breast cancer, before the start of systemic adjuvant treatment, and after adjuvant chemotherapy, in a large patient collective [15].

In order to understand the cause for CTC involvement, maturity, and outcomes, cytokine measurements in cancer therapy have become increasingly crucial [16]. As the involvement of the lymphatic system is known to play a key role in the progression of breast cancer [17], the aim of this study was the evaluation of Interleukin 15 (IL-15) and eotaxin as markers for CTC involvement in patients with the primary diagnosis of breast cancer. Cytokines and chemokines, through their tumor-promoting or tumor-suppressive properties, leading to the expression of either pro-inflammatory or anti-inflammatory cytokines [18], are able to trigger cancer progression and are presumed to be an important prognostic factor in the presence of breast cancer tumors [19]. Helper T-lymphocyte maturation depends on the arrangement of cytokines and results in the stimulation or suppression of critical cell derivation [20, 21]. One such pro-inflammatory cytokine is IL-15. IL-15 has early been identified as a 14–18 kDa protein [22]. The IL-15 structure produced in humans is described as a four α-helical bundle cytokine, positioned on chromosome 4q31 [22]. IL-15 was found to practice its biological effects, exploiting two separate signalling pathways. One of these paths encloses the conventional binding and signalling through the IL-15 receptor α (IL-15Rα), β and γ chains [22, 23]. This pathway is of interest as it has been described to result in the up-regulation of natural killer cells and T-cell activity in the human immune system [23–25]. The studies concerning IL-15 so far have already focused on its role concerning tumor genesis and its effect on proliferation, invasion, and metastasis production. IL-15 has predominantly been investigated in haematological malignancies [26–28] and is associated with a range of inflammatory disorders [25, 29]. The expression of IL-15 has been described as a protective factor in tumor genesis and against tumor progression in certain publications [30, 31], but in advanced solid cancers IL-15 expression acts contrarily thus contributing to disease progression [32–35]. Recently, studies demonstrated that IL-15 detected in sera of patients could provide neoplastic cells with a selective growth advantage in inducting and promoting certain types of malignancies [28, 36]. In regards to haematological malignancies, Cario et al. implied that IL-15 may increase cellular proliferation and is associated with poor prognosis in regards to relapse-free survival [37]. In a similar study by Chow et al., the serum levels of IL-15 were increased significantly in cancer patients compared to healthy individuals [38]. Eotaxin on the other hand is known to selectively recruit eosinophils, enhancing anti-tumor effects [39, 40].

The inconsistency of increased IL-15 levels acting both as a positive or negative marker in regard to tumor progression and survival outcome, could be explained by the role of dendritic cells (DCs) and their effect on the Immune system. DCs are known to be one of the most potent types of antigen-presenting cells in the human body and are involved in the regulation of both distinctive and adaptive immune responses [41]. DCs could therefore be of major importance concerning the effectiveness of IL-15 and its influence on survival outcome, and should be considered for future studies.

As no specific characteristics were described so far in relation to breast cancer, our goal was to examine the value of IL-15 in the T-lymphocyte immune response and the chemokine eotaxin, to reveal differences in the presence or absence in patients with breast cancer of the SUCCESS study; examining this with respect to CTC involvement, histopathological grading, lymph node status, hormone receptor type, OS, and DFS.

Detection of certain cytokine profiles and evaluation of their features can contribute to our better understanding of the disease, customized treatment options, and improved therapy observation. The tumor cell features in relation to cytokine profiles, their effect on the autoimmune response and changes in the microenvironment at the homing site, are of major importance for the future and therefore could be of use in generating new therapies.

## Materials and methods

### SUCCESS study design

SUCCESS was a prospective, randomized adjuvant study comparing three cycles of fluorouracil-epirubicin-cyclo-phosphamide (FEC; 500/100/500 mg/m^2^) followed by 3 cycles of docetaxel (100 mg/m^2^) every 3 weeks vs. three cycles of FEC followed by 3 cycles of gemcitabine (1000 mg/m^2^ d1,8)-docetaxel (75 mg/m^2^) every 3 weeks. After the completion of chemotherapy, the patients were randomized a second time to receive either 2 or 5 years of zoledronate. Hormone receptor–positive women, moreover, received applicable endocrine treatment.

The translational research questions related to CTC analysis, the blood sampling time points, and the methodology, were accordingly designed, and the prognostic value of the CTCs was described as a scientific objective of the study protocol. Eligible patients were defined as women with breast cancer (Lymph node positive subgroup and Lymph node negative subgroup with high risk traits including grade 3 tumor, hormone receptor negative, age under 35, ≥ pT2) who agreed to participate in the SUCCESS study (www.success-studie.de). The study was permitted by 37 German ethical boards (lead ethical board: LMU, Munich) and conducted in agreement with the Declaration of Helsinki.

### Blood sample collection for CTC enumeration

Blood samples for CTC enumeration were collected after study inclusion from 2090 consecutive patients after complete resection of the primary tumor and before adjuvant chemotherapy and after written informed consent was acquired. Sixty-four patients were excluded because of test failure or a time intermission of more than 96 h between the blood collection and sample preparation. A follow-up evaluation after chemotherapy and before the beginning of endocrine or bisphosphonate treatment was available for a subgroup of 1492 patients (see homepage: https://www.success-studie.de).

The method was conducted as described by the SUCCESS Study group [15]. CTCs were investigated using the CellSearch System (Veridex, Jansen Diagnostics, NJ, USA). Peripheral blood was drawn into three CellSave tubes (30 ml), sent at room temperature to the central laboratory at the University of Munich, and examined within 96 h of collection.

The patient samples were then centrifuged for 10 min at 800 × *g*. The plasma was removed, and a dilution buffer was supplemented. This combination was overlaid on 6 ml of Histopaque (Sigma, Steinheim, Germany) and centrifuged for 10 min at 400 × *g*. Subsequently, 7.5 ml of this sample enclosing the buffy coat were treated on the CellTracks AutoPrep system using the CellSearch Epithelial Cell Kit (Veridex). After immuno-magnetic enrichment with an antibody to Epithelial cell adhesion molecule (EpCAM), the cells were marked with fluorescent anti-cytokeratin (CK8,18,19–phycoerythrin) and anti-CD45 (CD45–allophycocyan) antibodies, and 4,6-diamidino-2-phenyl-indole-dihydrochloride was used to identify the intact cells.

### Detection of CTCs

The identification and enumeration of CTCs were achieved using the CellTracks Analyzer II. CTCs were stated as nucleated cells lacking CD45 and expressing cytokeratin. All positive samples were assessed by two independent investigators. Samples with at least one CTC per 30 ml of blood were regarded as CTC-positive.

The blood of 84 persons with no clinical evidence of malignant disease was processed blind and used as a negative control. Four of these negative controls (4.9%) contained cells that fit the definition of epithelial cells and which could be interpreted as CTCs (one control had one, two controls had two, and one control had three epithelial cells).

### Patients included

In this study 200 patients of the SUCCESS study were included and assigned into two groups: 100 Patients were CTC-positive (Group 1) and the other 100 patients were CTC-negative (Group 2). These two groups were framed and investigated accordingly. Patients from, respectively, groups were matched into pairs of two according to histo-pathological grading, lymph node involvement, hormone receptor type, and TNM classification. Furthermore, patients were matched according to OS (survived patients vs. deceased patients) at the end of the follow-up period. Out of 200 patient samples that were investigated, 160 patients were still alive at last observation at end of therapy and 40 patients had died during therapy. The groups investigated included 98 patients with tumor graded G2 and 102 patients graded G3. Matching criteria of the patient collective did not allow participants with G1 tumors (see Table 1). Tumor stage of the anamnestic diagnosis was classified according to the TNM-classification, which was conducted according to the WHO System [42]. The matching of patients was performed according to the criteria at the time of primary diagnosis. The histo-pathological grading was classified according to the Bloom and Richardson system classification [43].

**Table 1:** Patient and tumor characteristics

Characteristics	Alive patients after follow-up° No. (%)	Deceased patients after follow-up° No. (%)
No. of patients	160	40
Age in years (mean ± SD)	54.2 ± 9.6	53.3 ± 10.1
CTC status		
CTC negative (CTC = 0#)	80 (50)	20 (50)
CTC positive (CTC ≥ 1#)	80 (50)	20 (50)
Tumour size		
pT1a-c	78 (48.7)	10 (25.0)
pT2-4	82 (51.3)	30 (75.0)
Lymph node status		
pN0 (node negative)	54 (33.8)	8 (20.0)
pN1 (1–3 axillary)	82 (51.2)	12 (30.0)
pN2 (4–9 axillary)	22(13.7)	16 (40.0)
pN3 (≥ 10 axillary)	2 (1.3)	4 (10.0)
Grading		
G2	88 (55.0)	10 (25.0)
G3	72 (45.0)	30 (75.0)
Hormone receptor status		
Negative	44 (27.5)	10 (25.0)
Positive	116 (72.5)	30 (75.0)
Her2-neu status		
Negative	28 (17.5)	30 (75.0)
Positive	132 (82.5)	10 (25.0)
Menopausal status		
Premenopausal	69 (43.1)	14 (35.0)
Postmenopausal	91 (56.9)	26 (65.0)
Primary operation		
Breast conserving	113 (70.6)	29 (72.5)
Mastectomy	47 (29.4)	11 (27.5)
Systematic therapy		
Chemotherapy-FEC-D	76 (47.5)	21 (52.5)
Chemotherapy-FEC-DG	84 (52.5)	19 (47.5)

°Median follow-up was 65 months (range 1–96 months)

*CTC circulating tumour cell, *FEC-D* fluorouracil-epirubicin-cyclophosphamide (500/100/500 mg/m^2^, FEC) followed by doxetacel (100 mg/mg^2^), *FEC-DG* fluorouracil-epirubicin-cyclophosphamide (500/100/500 mg/m^2^, FEC) followed by gemcitabine (1.000 mg/m^2^ d1,8)-doxetaxel (75 mg/m^2^)

^#^Per 30 ml of blood

### Follow-up and patient evaluation

The median follow-up was 65 months (range 1–96 months). All time-to-event intervals were measured from time of the primary diagnosis to the date of the event or the date of the last adequate follow-up in case no event was reported. Patient outcomes were examined in terms of both DFS and OS. The patients were monitored at the study sites at 3-month intervals for the first 3 years, followed by every 6 months thereafter. The follow-up involved clinical examination and symptom-driven analyses, if necessary, at each visit, and mammography every 6 months. The collected data were acquired from the electronic case record forms of the SUCCESS study [15].

### Cytokine determination

For the measurement of cytokines, a commercial enzyme-linked immunosorbent assay (ELISA) was used to screen the blood serum samples for IL-15 and eotaxin. The ELISA was preformed with recently developed multi cytokine/chemokine arrays acquired by Meso Scale Discovery^®^ (Rockville, MD, USA). We used anti-species MULTI-ARRAY 96-well plates for the development of a sandwich immunoassay. Each assay in the panel was verified individually for the Specificity by running single calibrators with single detection antibodies. Non-specific binding levels were less than 0.5% for all assays. The 10 spot MULTI-SPOT plates were pre-coated with capture antibodies on independent and well defined spots that allowed us to immobilize a primary capture antibody against our protein of interest—specific for one of each vascular marker. Standards and samples were added to the appropriate wells. A standard curve was furthermore run with each assay. We firstly added the blood serum, calibrator and control. Subsequently we incubated at room temperature with shaking for 2 h. After eliminating excess samples from the well with wash buffer, we added the detection (anti-target) antibody conjugated with electrochemiluminescent labels over the course of two incubation periods. During the incubation period, where time slots differed in each test, the target present in the sample bound to the capture antibody, which was immobilized on the working electrode surface by the anti-species antibody. Recruitment of the labelled detection antibody by the bound target completed the sandwich. After a second shaking incubation period (time differed for each test) a wash buffer was used to eliminate all the unbound enzymes, and a MSD Read Buffer was added to produce the suitable chemical environment for electrochemiluminescence. We then loaded the plate into an MSD instrument (MESO QuickPlex SQ 120) for examination where voltage applied to the plate electrodes caused the captured labels to emit light. The intensity of the emitted light presented a quantitative measure for the amount of protein of interest present in the sample [44, 45] (see homepage: www.mesoscale.com).

### Statistical analysis

Statistical analysis was accomplished using SPSS 24.0 (IBM Corp., Armonk, NY, USA). The outcomes collected were recorded and inserted into the SPSS database in the implied manner. We evaluated the relationship between IL-15 and eotaxin and each matching criterion (CTC-positive vs. CTC-negative; OS -survival vs. death; grade 2 vs. grade 3; lymph node involvement vs. no lymph node involvement; triple-positive- vs. triple-negative breast cancer; progesterone receptor-positive vs. progesterone receptor-negative; oestrogen receptor-positive vs*.* oestrogen receptor-negative; HER2/neu receptor-positive vs. HER2/neu receptor-negative) using the non-parametric Spearman correlation coefficient. Each parameter to be considered was required to have a *p*-value of less than 0.05. Statistically significant results for the Spearman correlation coefficient were then assessed with the non-parametric Mann–Whitney U rank-sum test. Moreover, variables were examined by the use of box-plot analysis. All statistical tests were considered significant at *p* < 0.05.

## Results

### CTC-positive vs. CTC negative patients

**Fig. 1:**
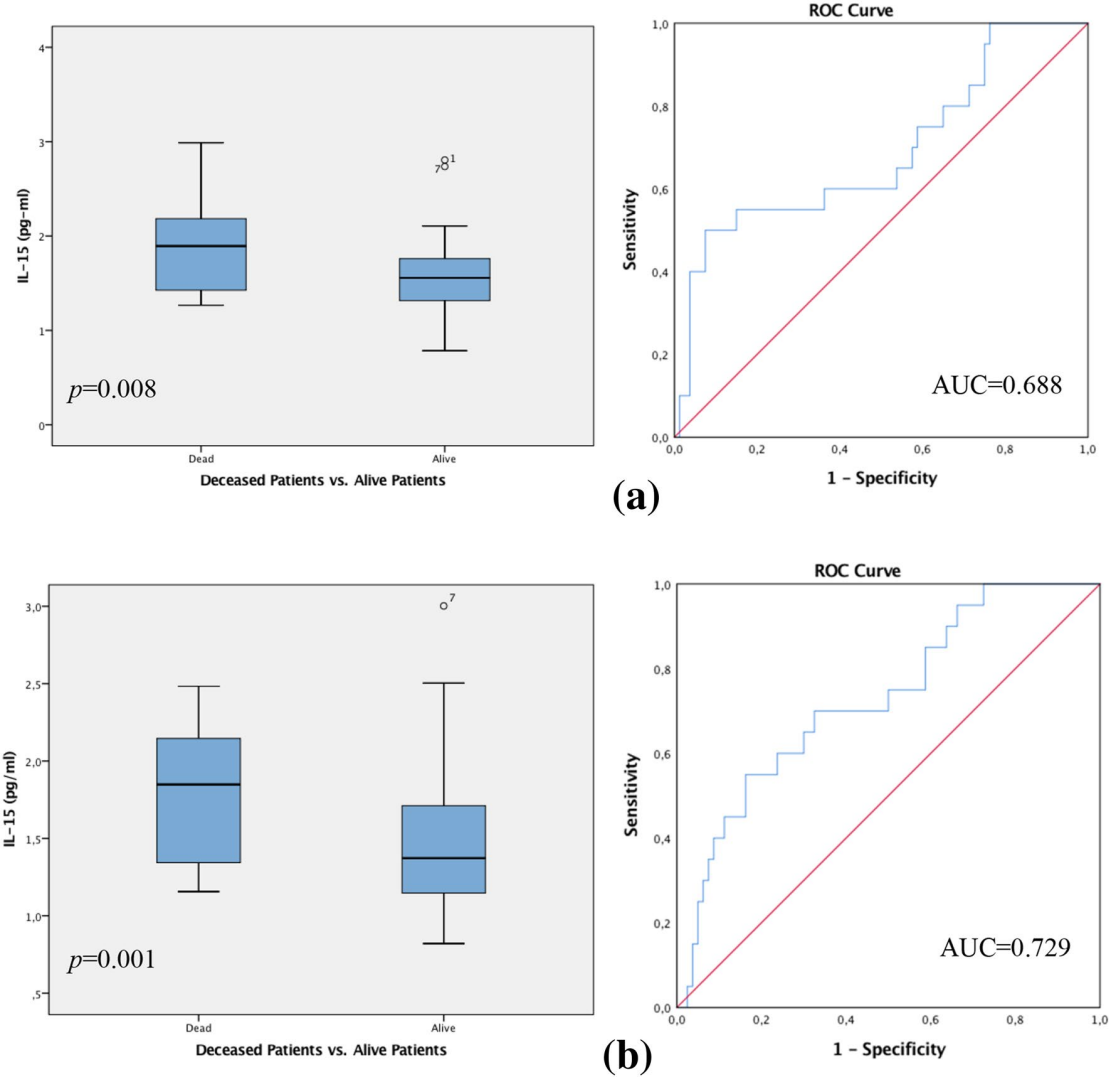
**a** Box plot analysis of Interleukin 15 expression (IL-15) in the sera of patients with CTC-*negative* breast cancer. IL-15 release was significantly higher in patients who died compared to patients who were living at follow-up (*p* = 0.008). Receiver operator curve analysis of sensitivity versus specificity gave an area under the curve (AUC) of 0.688 (**a**). **b** Box plot analysis of IL-15 expression and patients with CTC-*positive* disease. IL-15 release was expressively higher in the patient collective who died in the follow-up period compared to the living patient group (*p* = 0.001). Receiver operator curve analysis of sensitivity versus specificity gave an AUC of 0.729. The range amongst the 25th and 75th percentiles is demonstrated by boxes with a horizontal line at the median. The bars display the 5th and 95th percentiles. Circles specify values more than 1.5 box lengths. Asterisks specify values (marked with a number) more than 3.0 box lengths from the 75th percentile

In the overall patient collective, statistically significant differences were shown for IL-15 values in regard to the CTC-status. Amongst the CTC-*negative* group, those patients who died during the follow-up period expressed significantly higher levels of IL-15 compared to those patients being CTC-*negative* who were living at follow-up. This cohort expressed low levels of IL-15. The Spearman correlation coefficient assessed the p-value of 0.008 which was moreover supported by the Mann–Whitney-*U*-Test *p* = 0.008. ROC analysis implemented an AUC value of 0.688 (see Fig. 1a).

Furthermore, analysis amongst the CTC-*positive* collective showed that patients who died during the follow-up period, displayed significantly higher levels of IL-15 in comparison to CTC-*positive* patients who were living at follow-up; revealing expressively low levels of IL-15. The Spearman correlation coefficient evaluated the *p*-value at 0.001 which was reinforced by the Mann–Whitney-*U*-Test *p* = 0.001. ROC analysis was executed, revealing an AUC value of 0.729 (see Fig. 1b).

### Lymph node involvement

**Fig. 2:**
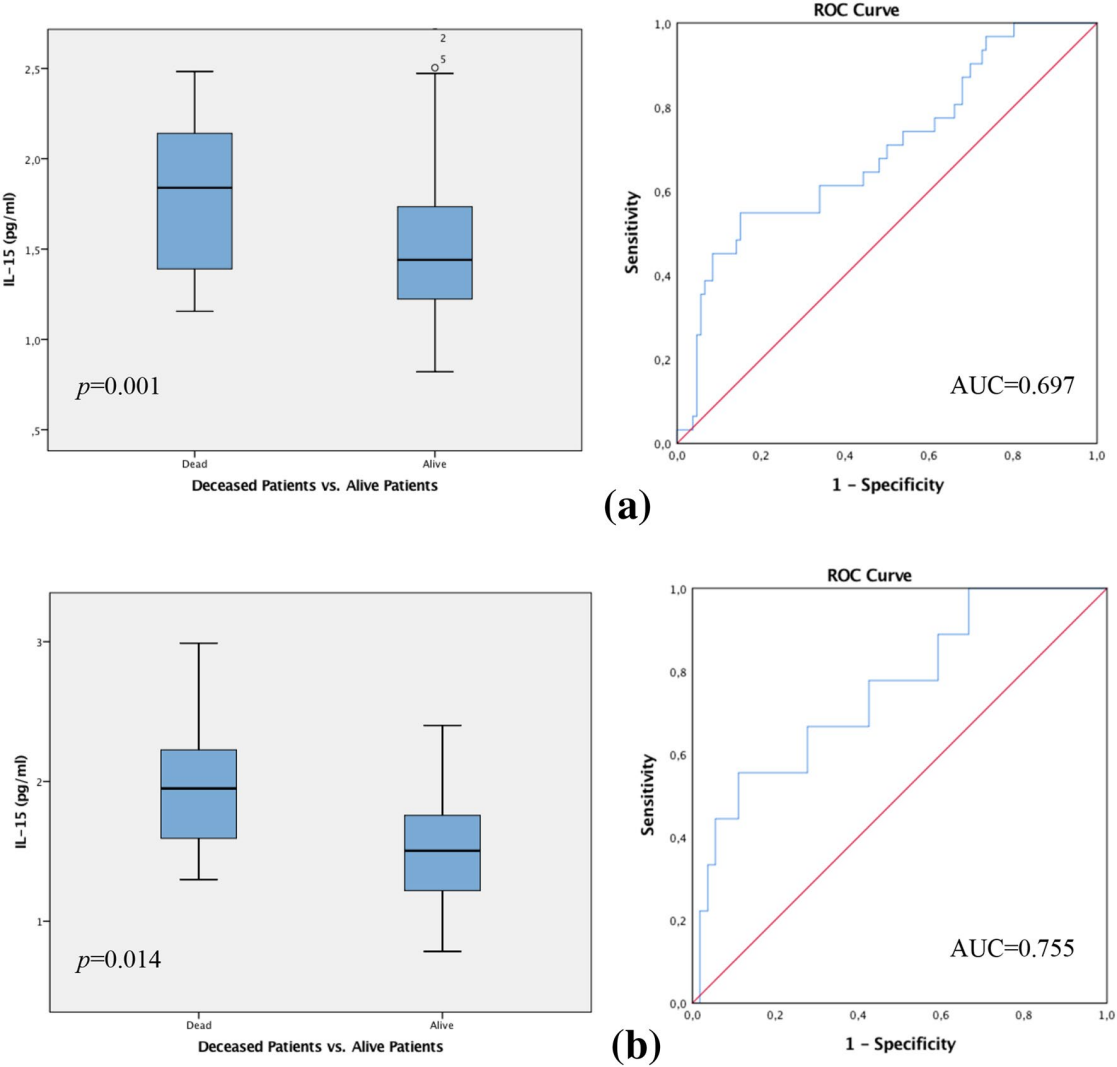
**a** Box plot analysis of IL-15 expression in the sera of patients with lymph node metastasis who died in the follow-up period, demonstrate considerably increased IL-15 values in contrast to the notable low IL-15 levels in patients with lymph node metastasis who were living up to follow-up (*p* = 0.001). **b** Box plot analysis showing the collective of patients with no lymph node involvement (N0) that died during the follow-up period. This group demonstrate significantly higher levels of IL-15 in contrast to the significantly decreased IL-15 levels in patients who remained alive (*p* = 0.014). The range amongst the 25th and 75th percentiles is demonstrated by boxes with a horizontal line at the median. The bars display the 5th and 95th percentiles. Circles specify values more than 1.5 box lengths. Asterisks specify values (marked with a number) more than 3.0 box lengths from the 75th percentile. Receiver operator curve analysis of sensitivity versus specificity gave an area under the curve (AUC) of 0.697 (**a**) and 0.755 (**b**), respectively

The patient collective with lymph node metastasis indicated statistically significant differences in regard to the patient survival outcome in correlation to IL-15 levels. Box-plot analysis showed that patients with lymph node metastasis who died during the follow-up period demonstrated considerably increased IL-15 values in contrast to the notable low IL-15 levels in patients with lymph node metastasis who were living at follow-up. The spearman correlation coefficient calculated the *p*-value at 0.001 which was furthermore confirmed by the Mann–Whitney-*U*-Test *p* = 0.001. ROC analysis assessed the AUC value at 0.697 (see Fig. 2a).

Moreover, a statistically significant correlation was also proven for the collective with no lymph node metastasis in association to the IL-15 levels and patient survival. The box plot analysis revealed that the collective of patients with no lymph node involvement (N0) who nonetheless died in the follow-up period, demonstrated significantly higher levels of IL-15 values in distinction to the notable low IL-15 levels in patients who remained alive. The Spearman correlation coefficient evaluated the *p*-value at 0.013 which was furthermore supported by the Mann–Whitney *U* Test *p* = 0.014. ROC analysis was executed, revealing an AUC value at 0.755 (see Fig. 2b).

### Hormone receptor status

**Fig. 3:**
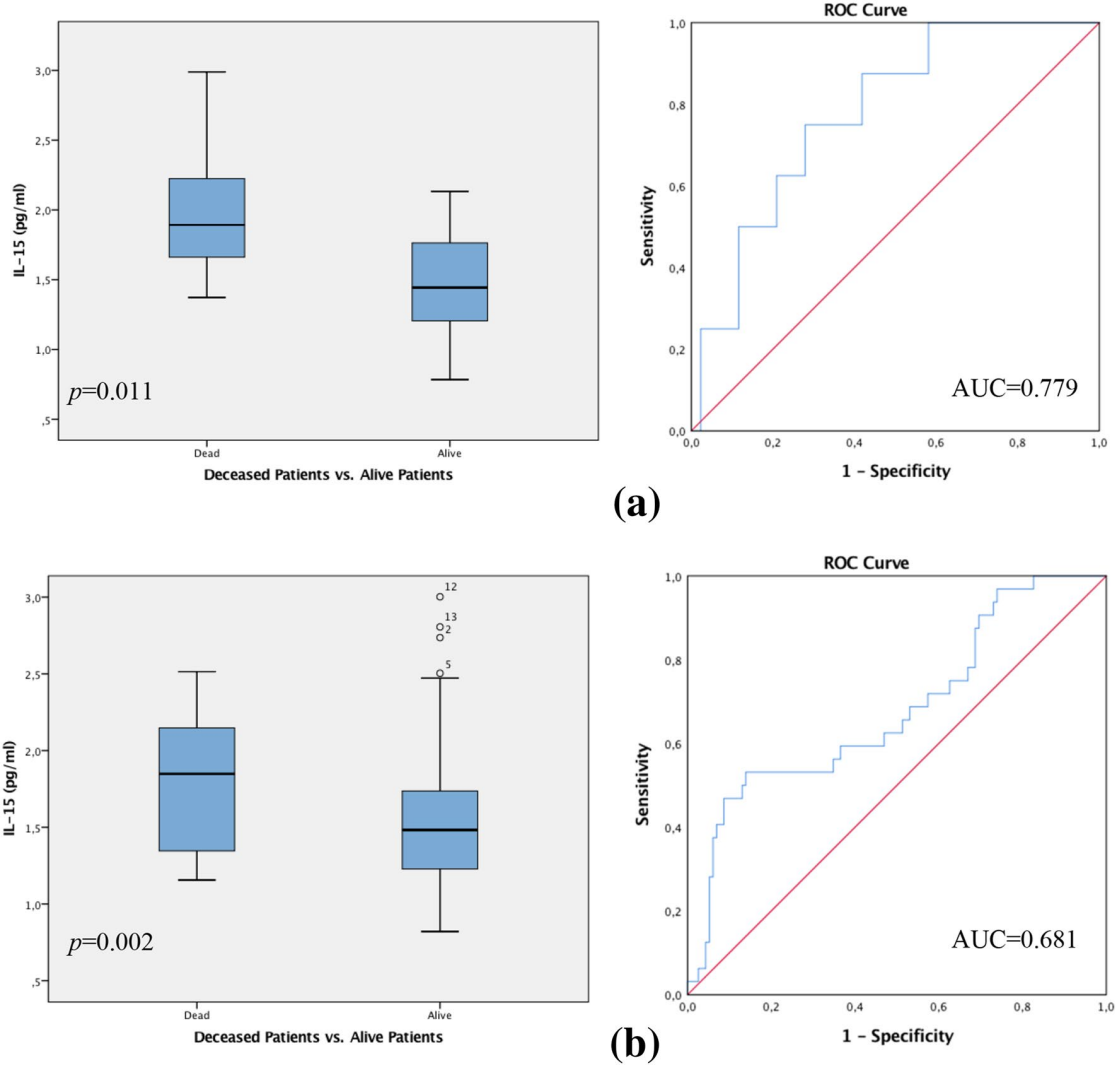
**a** Box plot analysis of IL-15 expression in the sera of patients with triple-*negative* breast cancer. Amongst this patient group, deceased patients expressed drastically higher levels of IL-15 compared to decreased IL-15 values in patients who remained alive (*p* = 0.011). **b** Similarly, we preformed a box plot analysis of IL-15 expression in the sera of patients with triple-*positive* breast cancer. Those patients with poor OS and DFS also displayed significantly higher levels of IL-15 in contrast to those patients who remained alive with a triple-*positive* breast cancer (*p* = 0.002). The range amongst the 25th and 75th percentiles is demonstrated by boxes with a horizontal line at the median. The bars display the 5th and 95th percentiles. Circles specify values more than 1.5 box lengths. Asterisks specify values (marked with a number) more than 3.0 box lengths from the 75th percentile. Receiver operator curve analysis of sensitivity versus specificity gave an area under the curve (AUC) of 0.779 (**a**) and an AUC of 0.681 (**b**)

In the total patient collective, statistically significant differences were shown for IL-15 values regarding the hormone receptor status. Patients with a triple-*negative* breast cancer (TNBC; progesterone receptor negative, oestrogen receptor negative, HER-2-neu receptor negative) who died during the follow-up period, expressed drastically higher levels of IL-15 as opposed to decreased IL-15 levels in patients who remained alive and showed a positive OS and DFS. The Spearman correlation coefficient assessed the *p*-value at 0.011, supported by the Mann–Whitney *U* Test *p* = 0.010. ROC analysis was executed, revealing an AUC value of 0.779 (see Fig. 3a).

Finally, patients with triple-*positive* breast cancer who died while showing poor OS and DFS also displayed significantly higher levels of IL-15, in contrast to those patients who remained alive with a triple-*positive* breast cancer. The latter showing significantly low IL-15 levels. The Spearman correlation coefficient disclosed a *p*-value of 0.002. Mann–Whitney-*U*-Test calculated *p* = 0.003. ROC analysis gave an AUC value of 0.681 (see Fig. 3b).

### Grading

**Fig. 4:**
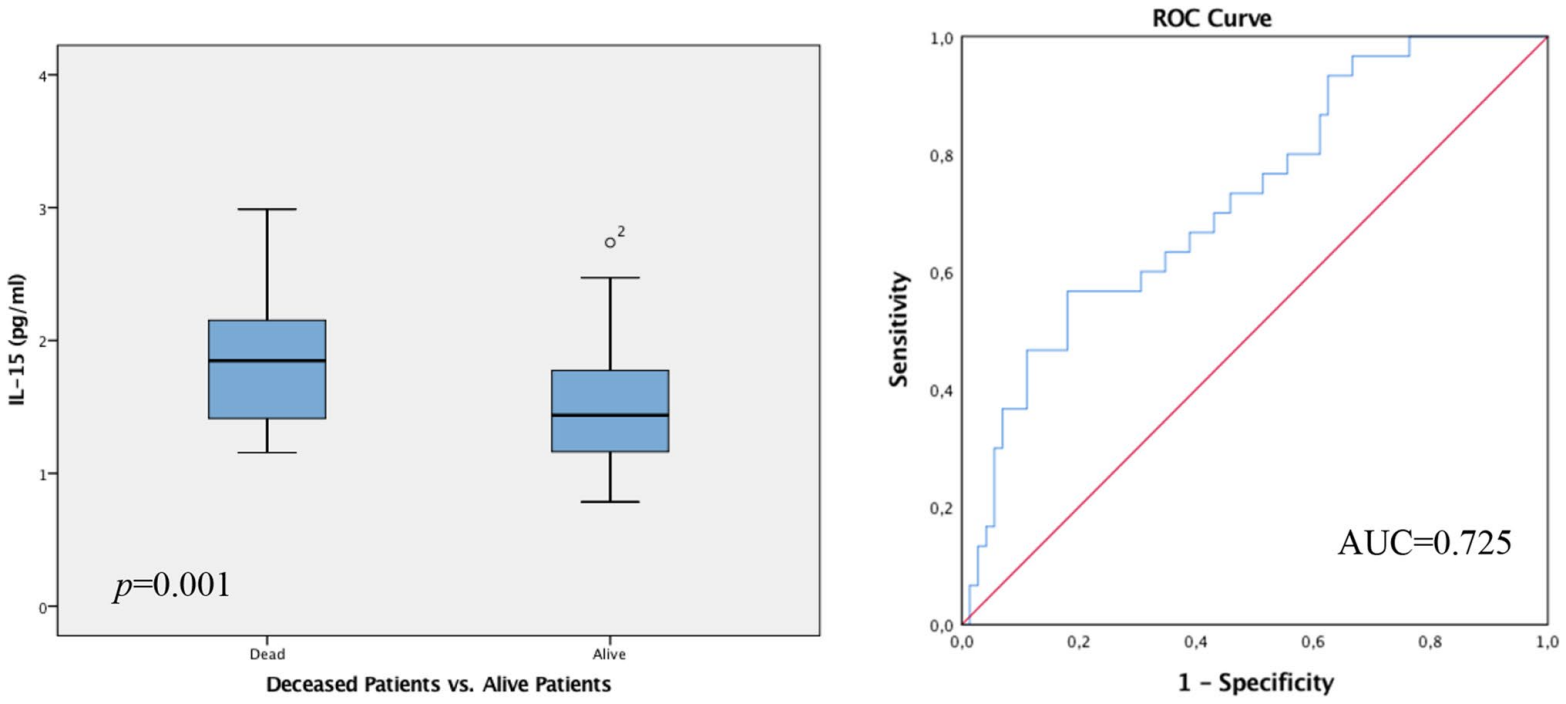
Box plot analysis of IL-15 expression in the sera of patients with G3 tumor. IL-15 release was significantly higher in the G3 patient collective who died during the follow-up period in contrast to low IL-15 levels in living patients at follow up with a G3 tumor (*p* = 0.001). The range amongst the 25th and 75th percentiles is demonstrated by boxes with a horizontal line at the median. The bars display the 5th and 95th percentiles. Circles specify values more than 1.5 box lengths. Asterisks specify values (marked with a number) more than 3.0 box lengths from the 75th percentile. Receiver operator curve analysis of sensitivity versus specificity gave an area under the curve (AUC) of 0.725

The data collected implies statistically significant differences concerning IL-15 levels in patients with a Grade 3 tumor (G3) in respect to OS and DFS. Patients with a G3 tumor, who died in the follow-up period, showed considerably increased IL-15 values, in contrast to the significantly decreased IL-15 levels in patients with G3 tumor with favourable OS and DFS and were living at follow-up. The Spearman correlation coefficient evaluated the *p*-value at 0.001, furthermore underlined by the Mann–Whitney-*U*-Test *p* = 0.001. ROC analysis revealed an AUC value of 0.725 (see Fig. 4).

### Patient survival

**Fig. 5:**
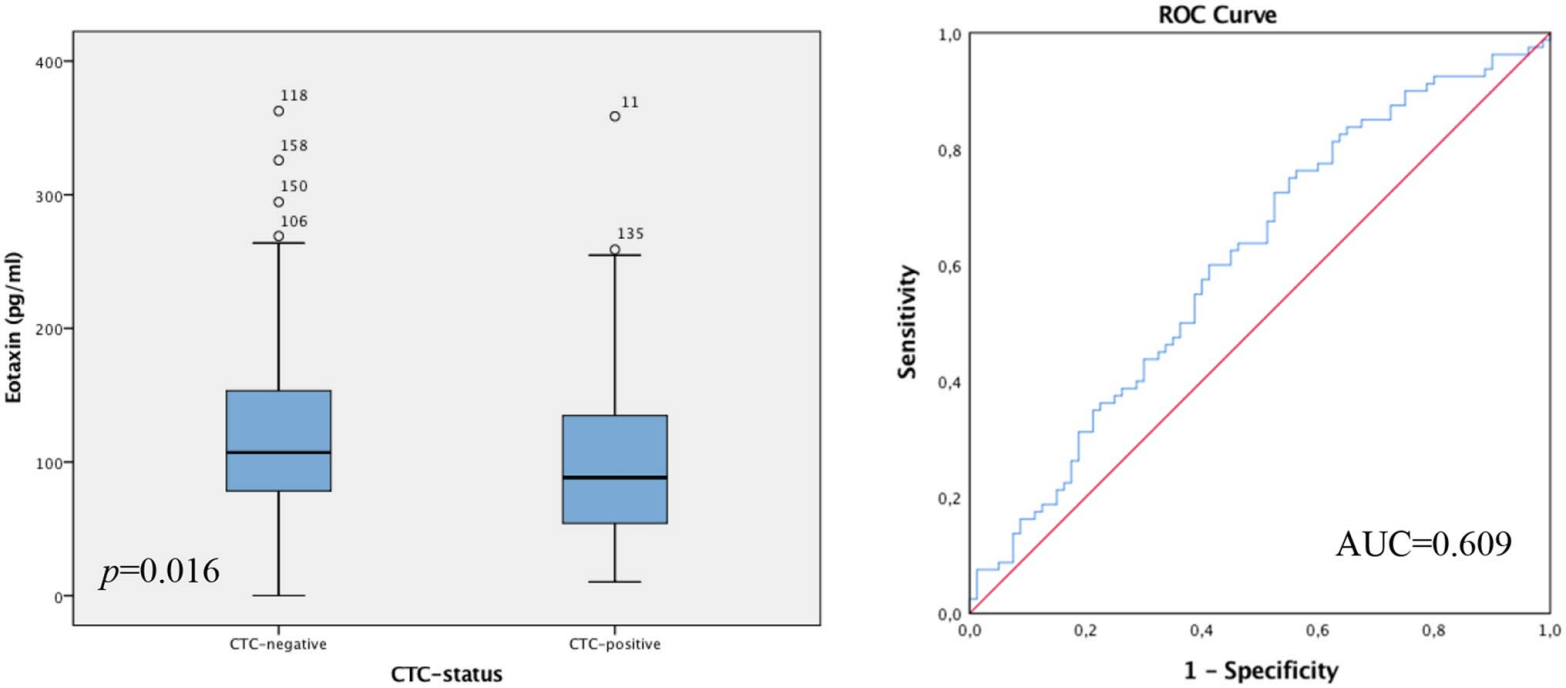
Box plot analysis of eotaxin expression in the sera of patients who remained alive up to follow-up. The patient group with the most favourable OS and DFS, furthermore CTC-*negative,* express increased eotaxin values compared to the reduced levels of eotaxin in the living patient group being CTC-*positive* (*p* = 0.016). The range amongst the 25th and 75th percentiles is demonstrated by boxes with a horizontal line at the median. The bars display the 5th and 95th percentiles. Circles specify values more than 1.5 box lengths. Asterisks specify values (marked with a number) more than 3.0 box lengths from the 75th percentile. Receiver operator curve analysis of sensitivity versus specificity gave an area under the curve (AUC) of 0.609

Analysing the patient collective who remained alive in the follow-up period in regards to patient survival, showed statistically significant differences in eotaxin values in correlation to the CTC status. The box-plot analysis revealed that the living patient collective, being CTC-*negative,* display higher levels of eotaxin compared to the reduced values of eotaxin in the living patient group, being CTC-*positive*. The Spearman correlation coefficient assessed the *p*-value at 0.017 which was also supported by the Mann–Whitney-*U*-Test *p* = 0.016. To continue, ROC analysis was performed, displaying an AUC value of 0.609 (see Fig. 5).

## Discussion

Within this study we analyzed the distribution of IL-15 and eotaxin concentrations and reveal the differences in their expression in the sera of breast cancer patients with and without circulating tumor cells. Prior studies have established the role of IL-15 in various tumor identities such as haematological malignancies [38, 46–48], colon cancer [32] or prostate cancer [49]. A clinical trial concerning adult acute lymphoblastic leukaemia even specified that higher IL-15 concentration was significantly associated with a poorer outcome and decreased survival rates compared to decreased serum concentrations of IL-15 in the control group of normal healthy individuals [48]. IL-15 levels are being discussed to be used as a specific tumor biomarker for prognostic values [28, 46]. Nevertheless, the prognostic role of IL-15 in breast cancer remains uncertain. In this study, we found that the serum concentration of IL-15 in breast cancer patients was significantly increased amongst those patients with poor OS and DFS and who had died at follow-up. In the overall patient collective, and regardless of their CTC-status, lymph node involvement or hormone receptor status, those patients with the poorest OS and DFS expressed significantly higher levels of serum IL-15 compared to those patients with the best survival outcome, which showed low IL-15 concentrations. Nevertheless, elevated serum concentrations of IL-15 significantly correlate to those patients with a Grade 3 tumor. Patients with poor OS who died during the follow-up period demonstrated considerably increased IL-15 values in contrast to the significantly low IL-15 levels in patients with a Grade 3 tumor and positive survival outcome. IL-15 values were also significantly decreased in patients with a Grade 2 tumor.

However, studies have indicated that IL-15 can lead to both a favourable and an unfavourable diagnosis, differing by the type of tumor. It is predicted that the inconsistent function of IL-15 in tumors lies within the different immune effects or single nucleotide polymorphism [46]. Rohena-Rivera et al. for example focused on determining the role of IL-15 in prostate cancer using in vitro and in vivo models by administrating IL-15 injections directly into the tumor tissue [49]. Blum et al. suggested that the presence of IL-15 caused inflammation, increased neutrophil infiltration, decreased the number of blood vessels, and was thus positively associated with biochemical recurrent-free survival in prostate cancer patients [50]. Nevertheless, these studies focused on the microenvironment of the tumor itself, suggesting that IL-15 expression, when found in the microenvironment, may provide a benefit for patients [49, 51]. Similarly, Kunivasu et al. defined IL-15 as a stimulus and attractant to Natural Killer T-cells (NK) and its expression is subsequently considered to be a protective trigger against the progression of certain tumor identities [30, 31, 35, 52]**.** Nevertheless, it has also been proven that in progressive tumors the infiltration of NK-cells into neoplasms suppressed the activity of the NK-cells [33, 34, 53]. Kunivasu et al. described IL-15 to have an opposite biological effect in colon cancer cells to that in the host immune system. IL-15 expressed on colon epithelial cells and cancer cells can promote the growth of cancer cells [23, 32, 54], while its stimulation on NK cells has an antitumor effect [35, 55]. Our study has shown that in this scenario both the IL-15-producing breast cancer cell and the patients’ host immune system producing IL-15, show a pro-tumor effect and promote tumor progression and metastasis. Our data indicate that when IL-15 can be detected in the sera of patients, its presence might contribute to the process of angiogenesis and progression of the disease thus resulting in a poorer survival outcome.

The association and exact mechanism of increased serum IL-15 levels and unfavourable prognosis are nevertheless not well defined so far. Numerous hypothetical justifications have however been proposed. IL-15 has been described to cause an increased invasion of inflammatory cells to the tumor site and proliferation of adipocytes that consequently cause an increase in size. In addition to the proliferation effect of IL-15, studies on the metastatic potential have shown an up-regulation of desmin and a-sma expression, both markers suggesting a stimulation to metastasis through IL-15 [49]. These results support our findings of increased IL-15 levels in deceased patients. IL-15 in in vitro and in vivo studies has been proven to increase the tumor volume as a consequence of inflammation and lipid mobilization [49, 56]. Wang et al. described the cell proliferation caused by IL-15, is mechanically induced by a strong proliferative signal by the JAK/STAT and Ras/MAPK pathway, and by increasing the anti-apoptotic proteins Bcl-2 and Bcl-Xl it can inhibit cell death [46, 57]. Furthermore, IL-15 decreases pro-apoptotic proteins such as BIM, PUMA by the activation of the PI3 K pathway [58–61]. Conversely, to reveal the exact biochemical and cellular function of IL-15 mediated stimulation in breast cancer, additional studies are needed.

IL-15 is a member of the 4 alpha helix group of cytokines, acting via its effective specific receptor IL-15Rα, that is expressed on antigen-presenting dendritic cells, monocytes and macrophages [62]. IL-15 demonstrates wide-ranging activity and can induce the differentiation and proliferation of T- B- and NK cells. IL-15 is furthermore known to stimulate the differentiation and immunoglobulin synthesis by B cells and has the ability to induce maturation of DCs [24, 62]. Studies have shown that DCs could cause this discrepancy of IL-15 levels acting both as a positive or a negative marker in regard to tumor evolution and survival outcome and its effect on the Immune system [63–65]. DCs, one of the most crucial components of the antigen-presenting stimulation pathway can have a massive impact on the regulation of anti-tumor immune responses [41]. DC based immunotherapies, thoroughly tested in liver cancer, are believed to contribute to the eradication of residual and recurrent tumor cells. It was suggested that mature DCs stimulated with OK432 produce large amounts of T helper type 1 (Th1) cytokines [66]. IL-15 is known to prime T lymphocytes and NK cells when secreted by DCs and to then stimulate anti-tumor immune responses [67, 68]. Studies have focused on enhancing tumor antigen presentation to T lymphocytes by transferring activated DCs with major histocompatibility, co-stimulatory molecules and loaded with tumor-associated antigens [69–73]. It has been suggested that transferred activated DCs result in higher concentrations of IL-15 and the chemokine eotaxin in the tumor, and collectively proposes that a DC-based, active immunotherapeutic strategy in combination with loco-regional treatment exerts beneficial anti-tumor effects against liver cancer and in DFS [63]. In contrast, it was reported that immature DC infused precisely into tumor tissues also contributed to the recruitment and activation of immune cells such as IL-15 in situ, however, this approach by itself generated limited anti-tumor effects due to probably insufficient stimulation of immature DCs [63, 74] and had no effect on prolonged recurrence-free survival. These studies therefore propose that the cytokine profile expressed by dendritic cells is dependent on the cell subtype and mode of activation whereas the concentration and presence of IL-15 itself does not correlate with a beneficial anti-tumor effect but indicates a close relation to the effectiveness of the DCs in regard to preventing tumor cell growth and survival.

On the other hand, eotaxin is known to selectively recruit eosinophils, act as a chemo-attractant, playing a major role in the inhibition of pro-angiogenetic factors, and thus resulting in an anti-tumor effect [39, 40, 75]. Eotaxin has been described to act as an important factor in the down regulation of angiogenesis by decreasing the stimulus for neo-vascularisation [76]. The development of invasive, aggressive and metastatic breast cancer is essentially reliant on the neo-vascularisation to provide blood supply for the nourishment and progress of the tumor. Accordingly, the increased eotaxin concentrations in the CTC negative collective could cause a significant interruption of tumor vascularization thus preventing CTCs being released into the peripheral blood by inhibiting circulation specific metastasis. A noteworthy delay in tumor growth could ensue in the absence of CTCs in those patients, subsequently enhancing survival and resulting in a favourable outcome.

The limitation of this study is the relatively short median follow-up period of 65 months. As previously described by Rack et al., the relative short follow-up period in the context of good prognosis results in minor absolute differences in the rate of recurrence and death. Further limitations are the lack of data on Ki-67 in the patient collective. This could be an advantage for more accurate subdivision of different breast cancer subtypes. The disproportional distribution of patient samples, considering 160 living patients, at last observation, and 40 deceased patients at the end of the follow-up period, could lead to limited statistical power. The limited sample size of 200 patients can be regarded as a limitation itself. It would be of interest to test for chemokine and cytokine levels in the overall collective of the SUCCES study group.

To sum up, accumulating evidence suggests IL-15 can initiate and promote certain types of malignancies. Nonetheless, an anti-tumour effect of IL-15 on the immune system has also been hypothesized in experimental trials [67, 68] and the activation of IL-15 is currently of major interest in several clinical Phase I trials [63, 74]. Tinhofer et al. have explored the expression patterns of the particularly sensitive IL-15 signalling pathway for multiple myeloma, a disease defined by the accumulation of malignant plasma cells in the bone marrow. Since IL-15 is also up-regulated in the sera of patients with multiple myeloma, it has been found that those malignant plasma cells expressed all three components of the IL-15R heterotrimer [26–28]. In contrast, normal B-cells from healthy donors downregulate IL-15Rα in response to IL-15. IL-15 overexpression in malignant plasma cells in in-vitro studies suggests a protection from spontaneous apoptosis and initiation of induced cell death [26–28, 38]. The data, similarly to our findings, imply that breast cancer and multiple myeloma cells are likely to reduce apoptosis and fortify themselves via autocrine IL-15 stimulation, thus becoming less dependent upon their microenvironment. In order to clarify the exact cellular mechanism of IL-15 resolved signalling in breast cancer, further studies are needed. Early trials in several solid tumors are showing significant clinical responses in patients who are treated with agents that block negative regulators of T cell activation [69–73]. Nonetheless it is suggested that chronic stimulation can lead to malignant transformation of T and NK cells and appear to encourage its oncogenic properties. Exploiting IL-15′s influential properties to improve lymphocyte effector function in the setting of malignancy is likely to become more structured in the future and might lead to a broader use in the treatment of tumor malignancies.

These findings for the first time demonstrate the functional interaction of increased IL-15 concentrations in the peripheral blood and poor OS and DFS, regardless of the CTC status, lymph node metastasis, or hormone receptor status. A close relationship of increased IL-15 levels in patients with a Grade 3 breast tumor and those with the poorest survival outcome were moreover confirmed. These outcomes imply a substantial function of IL-15 in the pathogenesis of breast cancer and thus could offer new insights into tumor evolution and possible therapeutic approaches. Serum IL-15, simply measured in the everyday clinical routine, could therefore be an independent prognostic marker of major importance in terms of DFS and OS for breast cancer patients. The immense impact of IL-15 as an independent marker for predicting the survival outcome is furthermore underlined by the fact that even in the patient collective with favourable tumor characteristics at primary diagnosis, such as CTC negativity, no lymph node involvement or triple-positive breast cancer, and increased serum IL-15 concentrations were significantly increased in those patients that deceased. Bearing this in mind, IL-15 could be used as an early marker to highlight high-risk patients and the subsequent adjustment of the cancer therapy strategy. In contrast, it can also prevent over treatment in those patients with decreased IL-15 levels. The increased levels of the chemokine eotaxin in CTC negative patients with the best OS and DFS outcome on the other hand, suggests that the overexpression in tumor cells inhibits CTCs entering the peripheral blood, thus emphasizing a significant anti-angiogenic effect, inhibiting tumor growth and metastasis. Furthermore, in regard to CTC negativity, the eotaxin value may potentially serve as a predictive marker.

## Abbreviations

CTC: Circulating tumor cells
ELISA: Enzyme-linked immunosorbent assay
TH1: T helper 1
TH2: T helper 2
Treg: Regulatory T cells
IL-15: Interleukin 15
DFS: Disease-free survival
OS: Overall survival
AUC: Area under the curve
FEC: Fluorouracil-epirubicin-cyclo-phosphamide
NK: Natural killer
DC: Dendritic cells
HER2: Human epidermal growth factor receptor 2
